# Non-alcoholic fatty liver disease is associated with dysregulated bile acid synthesis and diarrhea: A prospective observational study

**DOI:** 10.1371/journal.pone.0211348

**Published:** 2019-01-25

**Authors:** Richard N. Appleby, Iman Moghul, Shahid Khan, Michael Yee, Pinelope Manousou, Tracy Dew Neal, Julian R. F. Walters

**Affiliations:** 1 Division of Digestive Diseases, Imperial College London, London, United Kingdom; 2 Department of Diabetes and Endocrinology, Imperial College Healthcare NHS Trust, London, United Kingdom; 3 Department of Biochemistry, Kings College Hospital NHS Trust, London, United Kingdom; Medizinische Fakultat der RWTH Aachen, GERMANY

## Abstract

**Background:**

Non-alcoholic fatty liver disease (NAFLD) may be associated with changes in bile acid (BA) metabolism. Hepatic BA production, measured by serum levels of the precursor 7α-hydroxy-4-cholesten-3-one (C4), is regulated by the farnesoid-X-receptor (FXR)-dependent ileal hormone fibroblast growth factor 19 (FGF19). Low FGF19 and high C4 are features of chronic BA diarrhea. Obeticholic acid, an FXR agonist, stimulates FGF19 and has shown therapeutic potential in both BA diarrhea and in NAFLD. We hypothesized there are associations of FGF19, C4 and BA diarrhea with NAFLD.

**Methods and findings:**

127 patients with known NAFLD were recruited prospectively. Clinical features, including metformin use, markers of NAFLD severity and BA synthesis were analyzed. The overall incidence of chronic diarrhea was 25%, with features of BA diarrhea in 12%. FGF19 negatively correlated with C4 (r_s_ = -0.43, p = 0.001) and with alanine aminotransferase (r_s_ = -0.22, p = 0.03), but not with either NAFLD fibrosis or Fibroscan scores. High C4 was associated with a higher NAFLD fibrosis score (p < 0.05), and with diarrhea (p = 0.001). The median NAFLD fibrosis score was higher in those with diarrhea (p = 0.002). Metformin use, in 44% overall, was particularly associated with diarrhea (in 36% vs 17%, p = 0.02), and a lower median FGF19 (74 vs 105 pg/mL, p < 0.05).

**Conclusions:**

Increased hepatic BA production and diarrhea, but not low FGF19, were associated with increased NAFLD fibrosis score, indicating dysregulation of the FXR-FGF19 axis and suggesting hepatic FGF19 resistance. Metformin use was an important factor in a subgroup, lowering FGF19, and resulting in bile acid diarrhea.

## Introduction

Non-alcoholic fatty liver disease (NAFLD) affects 30–40% of the Western population. It comprises a spectrum from benign hepatic steatosis, with 30% progressing to inflammatory non-alcoholic steatohepatitis (NASH), and 3% progressing to fibrosis and cirrhosis.[1] Insulin resistance, dyslipidemia and obesity are important risk factors for the development of NAFLD and are part of the metabolic syndrome. Many NAFLD patients are on treatment for these conditions, including anti-diabetics such as metformin, statins or other medications.[2]

Fibroblast growth factor 19 (FGF19) is an endocrine fibroblast growth factor released by the ileum into the portal circulation in response to bile acid (BA) stimulation of the nuclear receptor farnesoid X receptor (FXR). The principal action of FGF19 is the inhibition in hepatocytes of CYP7A1, the rate limiting enzyme of BA synthesis by the classical pathway.[3] CYP7A1 activity can be measured by quantification of the BA precursor 7α-hydroxy-4-cholesten-3-one (C4) in the serum. C4 has a strong inverse correlation with FGF19.[4] FGF19 produces its hepatic effects via FGF-receptor 4 (FGFR4) and the co-receptor beta-Klotho(KLB), creating an FXR-FGF19-FGFR4/KLB axis of BA regulation.

FGF19 has several other metabolic actions apart from the control of BA synthesis. Lower FGF19 levels are found in obesity and an increase in FGF19 after Roux-en-Y gastric bypass surgery is associated with remission of type 2 diabetes.[5, 6] In NAFLD, a study of 15 adults with NAFLD reported normal baseline and post-prandial serum FGF19 levels in patients with NAFLD, but no reduction in C4 after a meal, indicating hepatic resistance to FGF19 as a possible factor, as was also shown for insulin resistance.[7] Lower FGF19 was associated with biopsy proven NAFLD in 91 patients and a more severe histology ballooning score.[8] Raised serum C4 was found in NASH (but not NAFLD) and associated with increases in specific BA, reduced fecal bacteria and with low FGF19, in a study totaling 53 subjects.[9] Fecal BA were increased in NASH. Another study on a total of 27 patients with NASH and controls (mean age about 13) recently reported raised serum and fecal BA, without a significant increase in C4, although serum FGF19 was lower.[10]

Studies in another pediatric NAFLD population showed a stronger association with low FGF19. A study of 23 obese adolescents showed that low FGF19 was associated with insulin resistance, and inversely correlated with alanine aminotransferase (ALT) and triglycerides.[11] Low FGF19 was associated with NALFD (median FGF19 was 81 vs. 201pg/mL in controls), and was even lower in those with progression to NASH (54pg/mL).

The FXR-FGF19 axis has been identified as a therapeutic target in NAFLD. Treatment with the synthetic FXR agonist obeticholic acid improved the homeostatic model assessment insulin-resistance (HOMA-IR), a measure of insulin resistance, in patients with NAFLD.[12] The phase 2 FLINT trial of OCA in NAFLD was stopped early when it showed a clear benefit of OCA, with improved steatosis, lobular inflammation and fibrosis scores on repeat biopsy in OCA treated patients compared to placebo.[12] Treatment with an engineered FGF19, NGM282, has also shown promise in NASH.[13]

Low FGF19 is also associated with bile acid diarrhea (BAD); the lack of suppression of CYP7A1 causes an overproduction of BAs that spill over into the colon producing diarrhea.[14] The primary form of BAD, without a secondary ileal defect such as resection or inflammation, has a prevalence of 1% in the general population and at least 25% in patients with diarrhea-predominant IBS (IBS-D).[15] It can be readily diagnosed by the ^75^selenium homocholic acid taurate (SeHCAT) test where this test is available. This measures retention (and hence loss) of the radiolabeled modified bile acid. Retention of <15% after 7 days is diagnostic of BAD. SeHCAT retention correlates inversely with fecal BA.[16] An alternative approach to diagnosis is to measure serum C4 or FGF19. These tests have not been standardized and are not widely available. C4 values above 28-30ng/mL (70nmol/L) have been used to diagnose BAD;[17] a raised C4 of >48ng/mL (120nmol/L) has been quoted to have a sensitivity of 90% and a specificity of 79% to detect a SeHCAT <15%.[18] We found that FGF19 <145pg/mL has a negative and positive predictive value of 82% and 61% to detect a SeHCAT <10%.[19]

We recently reported a retrospective analysis of over 500 patients with chronic diarrhea who had all undergone SeHCAT testing. We found an increased incidence of high ALT and steatosis on imaging in those who had SeHCAT retention <15% (Odds Ratio 2.5).[20] In the study of patients with NAFLD reported here, we hypothesized that they would show abnormalities in the FXR-FGF19 axis, such as an increased incidence of raised C4 and low FGF19, and that by association, there would also be an increased incidence of bile acid diarrhea.

## Methods

### Subjects

This study was performed with ethics committee approval (15/EM/0082). The standards of Good Clinical Practice for research were followed with all subjects giving fully informed written consent. Patients with NAFLD were recruited prospectively from the NAFLD clinic at St Mary’s Hospital between February and September 2015. These were patients with an existing diagnosis of NAFLD who had been referred to tertiary care by GPs or secondary care specialists. The diagnosis of NAFLD was made by presence of hepatic steatosis on ultrasound in the absence of another liver disease on blood testing (viral hepatitis markers, autoantibodies, serum ferritin/ total iron binding capacity) or alcohol consumption >21 units/week. All patients in the clinic had routine measurements of body mass index (BMI), fasting blood tests and transient elastography (Fibroscan). If the Fibroscan revealed potential hepatic fibrosis (Fibroscan result >6kPa), patients were offered a liver biopsy.

Consecutive patients aged 18–80 were recruited, and only those with other potential causes of liver disease, chronic diarrhea of another etiology, or were unable to consent, were excluded. The study aimed to recruit 124 patients to give a power of 80% to detect an incidence of BAD of 2.5% with an α of 0.05 (as predicted by the retrospective study).

### Data collection

After consenting to participate, the patient’s demographics, brief medical and drug history were collected. A single fasting blood test was performed for FGF19, C4 and genomic DNA. If patients were not fasting, they were asked to return for a fasting sample. In addition the following data was collected from the medical notes; BMI, ALT, AST, fasting lipid profile and glucose, Fibroscan score and liver biopsy results.

### Identification of patients with diarrhea

On the initial visit each patient was asked the question: *‘Have you experienced diarrhea for more than 1 week in the last 3 months*?’ If the patient said yes, they were given a daily symptom diary to record for 7 consecutive days. They recorded the frequency and Bristol stool form scale of each stool (a validated surrogate of GI transit time).[21] After 7 days, the diaries were collected, and if the total number of stools was ≥21 stools/week, with 50% being types 5, 6 or 7, the patient was asked to attend the gastroenterology outpatient clinic, where investigations appropriate to the patient’s presentation, as determined by the clinician, were performed.

All patients who reported diarrhea were defined as having chronic diarrhea. Patients with diarrhea having a FGF19 <145ml/ml and C4 >70nmol/L were defined as having bile acid diarrhea, as shown previously.[4, 17, 19, 22]

### Blood sample processing and storage

Blood samples for FGF19 and C4 were placed immediately on ice until centrifugal sera separation and storage at -80°C within 2 hours of collection. Blood for DNA was frozen at -20°C until extraction.

Sera were transferred to the biochemistry lab at Kings College Hospital in batches. FGF19 was measured in triplicate by a commercially available ELISA kit (Quantikine ELISA, R&D systems Europe, Abingdon, UK) and the mean value reported in pg/mL. A value below 145pg/mL was defined as low.

C4 was measured by Tandem (liquid/gas phase) chromatography (LC-MS/MS). The assay is locally calibrated by Kings College laboratory between the values of 2.5–1000 nmol/L. A value above 70nmol/L was defined as high. Combined FGF19 <145pg/mL and C4 >70nmol/L was the definition of dysregulated FGF19 axis.

### NALFD severity assessments

#### NAFLD fibrosis score

The NAFLD fibrosis score for each patient was calculated for each patient using the equation: -1.675 + 0.037 × age (years) + 0.094 × BMI (kg/m2) + 1.13 × IFG/diabetes (yes = 1, no = 0) + 0.99 × AST/ALT ratio– 0.013 × platelet (×109/l)– 0.66 × albumin (g/dl).

A score of < -1.455 has a negative predictive value of 93% for fibrosis and >0.675 has a positive predictive value of 90% for fibrosis.[23]

#### Fibroscan

Transient hepatic elastography was measured by Fibroscan 502 with medium or XL probes if BMI>30 (Echosens, Paris, France) for all patients prior to enrolment and recorded at the first visit. Fibroscans were performed by specialist nurses who have performed over 50 procedures. Individual scores were reported in kPa and were the median of 10 valid measurements, with a 60% success rate and an interquartile range of less than 30%. A Fibroscan score >7.9kPa has been shown to predict fibrosis score on histology with a sensitivity and specificity of 91% and 75% in NAFLD.[24]

#### Liver histology

Liver histology reports were collected for those patients who had undergone liver biopsy within the last 2 years. The report was categorized into ascending levels of severity according to the wording: steatosis, steatohepatitis, fibrosis or cirrhosis.

### Statistical analysis

Correlations were calculated FGF19 and C4 values against NAFLD fibrosis score, ALT and Fibroscan score. Demographic data was assessed for variance using ANOVA, a subgroup analysis of those factors identified as significant on the ANOVA was be performed. The odds ratio of chronic diarrhea in patients with NAFLD was calculated and the variance assessed by Fisher’s exact test. Non-parametric data was assessed for variance using a Mann-Whitney U test. All analyses were performed using Prism v6.0 (Graphpad, La Jolla, CA).

## Results

A total of 127 patients were enrolled into the study. Blood tests for FGF19 and C4 were obtained on 96 patients as 31 patients did not provide a fasting sample. Fibroscan was performed on 78 patients, and 50 had a liver biopsy. Diarrhea was reported in 32 subjects (25%), 25 of whom had blood tests for FGF19 and C4, 25 had Fibroscan and 18 had liver biopsy.

### Demographics

Demographics are shown in Table 1. The incidence of diarrhea was significantly raised in females, those with a BMI>25, white ethnic groups, diabetics and those taking metformin. These groups were selected for subgroup analysis.

**Table 1:** Demographics of study cohort divided by presence or absence of chronic diarrhea.

		All	No Diarrhea	Diarrhea	p
		(n = 127)	(n = 95)	(n = 32)	
**Age (years)**	Mean	52.51	52.28	53.18	0.74
	Range	18–83	18–76	27–83	
**Female (%)**		42 (33)	17 (18)	25 (78)	0.009
**BMI kg/m^2^**	Mean	30.84	29.9	33.5	0.0007
	Range	20.9–47.8	20.9–42.9	21.2–47.8	
**(% >25/>30)**			(91/54)	(97/66)	
**White ethnicity (%)**		54	51	76	0.02
**Asian ethnicity (%)**		27	33	14	0.06
**Black ethnicity (%)**		6	9	7	1
**Far Eastern ethnicity (%)**		2	3	0	1
**Diabetes (%)**		45	39	63	0.02
**Cholecystectomy**		6	3	3	0.14
**Alcohol (units/week)**		2.26	2.47	1.63	0.41
**Metformin (%)**		44	38	63	0.02
**Insulin**		11	9	19	0.12
**GLP-1 Agonist**		4	2	9	0.10
**Sulfonylurea**		17	13	4	1
**Glitazone**		5	4	1	1
**SGLT-2 Inhibitor**		2	1	1	0.44
**DDP Inhibitor**		8	5	3	0.42
**Orlistat (%)**		5	5	3	0.58
**Statin (%)**		51	56	47	0.68
**Fibrate**		6	6	0	0.34
**Exetimibe**		2	1	1	0.44

### Severity of NAFLD, FGF19 and C4

FGF19 and C4 values are shown in Table 2. FGF19 was <145pg/mL in 62 patients and C4 was >70nmol/L in 24. FGF19 <145pg/mL and C4 >70nmol/L (our definition of a dysregulated FGF19 axis) was present in 19 patients (20%). Overall, FGF19 had a negative correlation with ALT (r_s_ -0.22, p = 0.03), as did C4 and ALT (r_s_-0.18, p = 0.04). As expected, FGF19 correlated negatively with C4 (r_s_ -0.43, p<0.001) but neither FGF19 or C4 correlated significantly with the NAFLD fibrosis score or Fibroscan score (Table 3). FGF19 was not significantly associated with bilirubin.

**Table 2:** Serum FGF19 and C4 values in NAFLD patients overall and with or without diarrhea.

		All	No Diarrhea	Diarrhea	p
		(n = 96)	(n = 71)	(n = 25)	
**FGF19 (pg/mL)**	Mean	131.3	138.7	110.4	
	SEM	12.5	15.7	17.5	
	Median	86.8	95.2	73.0	0.23
	IQR	63.4–186.9	64.4–184.6	59.0–138.8	
**C4 (nmol/L)**	Mean	53.8	43.9	82.0	
	SEM	4.1	3.6	10.0	
	Median	44.7	35.4	74.9	0.0002
	IQR	25.8–72.5	23.3–56.2	42.2–113.5	

Means ± SEM, medians and inter-quartile ranges (IQR) are shown. FGF19 and C4 values in the No diarrhea and Diarrhea groups were compared with Mann-Whitney U-tests.

**Table 3:** Correlation coefficients between indicators of bile acid disequilibrium and markers of NAFLD severity.

	ALT	NAFLD Fibrosis Score	Fibroscan
**FGF19**	-0.22* (n = 98)	0.03 (n = 98)	-0.014 (n = 80)
**C4**	-0.18* (n = 98)	0.14 (n = 98)	0.04 (n = 80)
**Stools/week**	0.22 (n = 23)	-0.24 (n = 23)	0.07 (n = 23)

*p<0.05, Spearman’s rank correlation.

**Fig 1:**
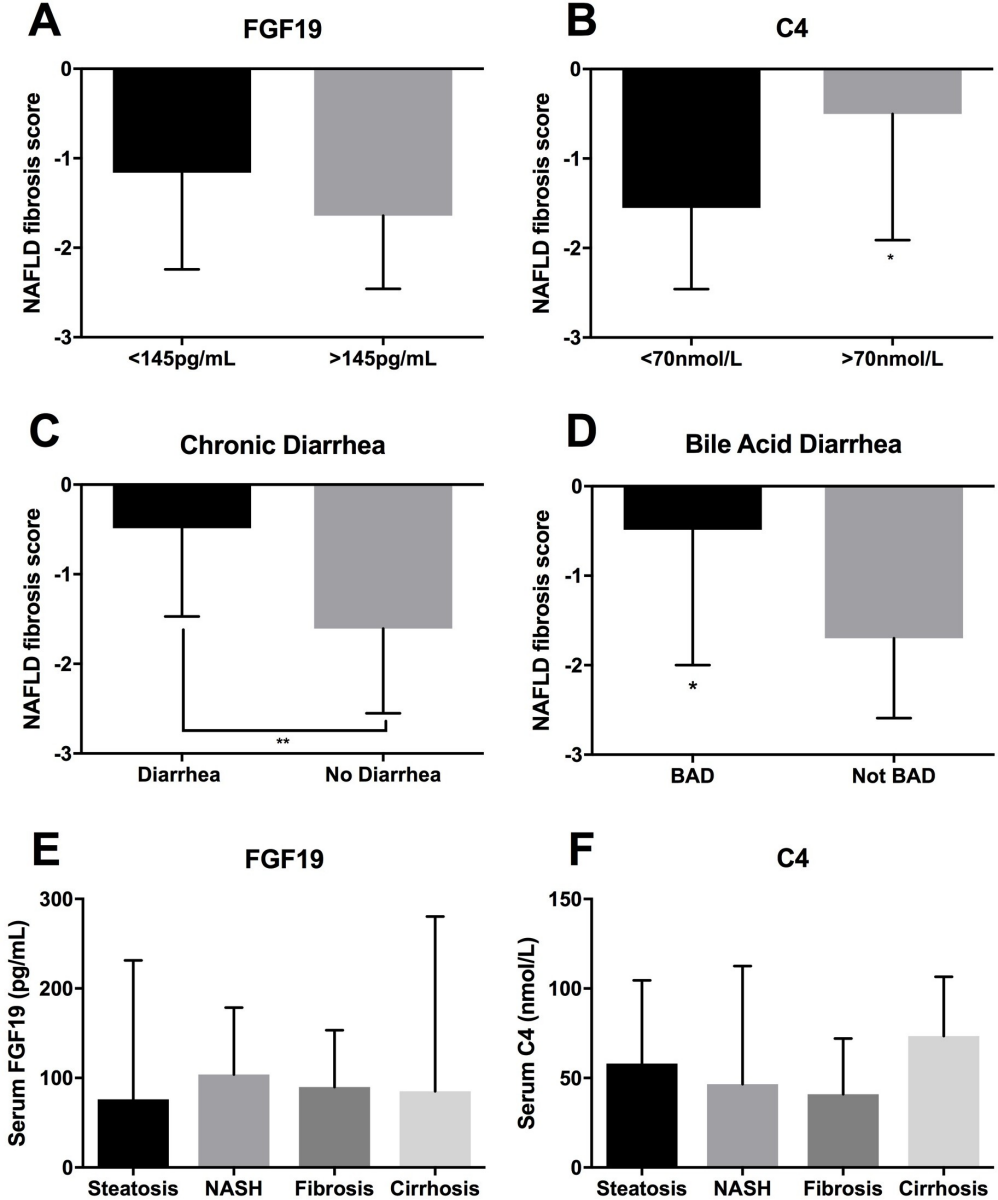
NAFLD fibrosis scores in different groups, and FGF19 and C4 by liver biopsy findings. Median (IQR) NAFLD fibrosis score by FGF19 (A), C4 (B), presence of chronic diarrhea (C) or bile acid diarrhea (D). Median (IQR) FGF19 (E) and C4 (F) by liver biopsy findings. NASH: Non-alcoholic steatohepatitis. *p<0.05 **P<0.01.

The median NAFLD fibrosis score was -1.27 (IQR -2.37 to -0.4). As shown in Fig 1A–1D, this was significantly higher for patients with C4>70nmol/L (-0.5; IQR -1.74 to 0.5), patients with dysregulated FGF19 axis (-0.49; IQR -1.53 to 0.14) and patients with diarrhea (-0.54; IQR -1.77 to 0.12) than those without these features. Differences were not significant for FGF19 <145pg/mL (-1.1; IQR -2.14 to -0.25) or the defined BAD group (-0.49; IQR -1.61 to -0.18).

Median Fibroscan score was 6.8kPa (IQR 5.4–9.6). There were no significant differences between the median values of those with C4>70nmol/L (7.9; IQR 6.2–12), FGF19<145pg/mL (7.15; IQR 5.5–10.4), dysregulated FGF19 axis (7.4; IQR 5.8–11), diarrhea (7.3; IQR 5.4–11.5) or BAD (6.9; IQR 5.9–8.4).

Biopsy results were available in 50 patients with FGF19 and C4 data. Histology was reported in 7 as steatosis, 7 steatohepatitis, 29 fibrosis and 7 as cirrhosis. Kruskal-Wallis tests of median FGF19 and C4 values showed no significant variation between the groups (FGF19 0.27, p = 1; C4 1.8, p = 0.6; Fig 1E and 1F). The lowest median FGF19 value was in steatosis (76pg/mL; IQR 58.9–168.4) and the highest was for steatohepatitis (103.9pg/mL; IQR 70.5–187.9). The highest median C4 was for cirrhosis (73.4nmol/L; IQR 28.2–99.9) and lowest for fibrosis (40.9; IQR 25.9–69.3). There were no differences of histological stage in patients with dysregulated FGF19 axis, chronic diarrhea or BAD.

### Incidence of chronic diarrhea and bile acid diarrhea

The incidence of chronic diarrhea was 25% in the whole cohort and 26% in those who returned for FGF19 and C4 testing; 18 of these subjects (75%) returned the stool diary. The overall incidence of bile acid diarrhea (defined as chronic diarrhea with raised C4 and low FGF19) was 12% (n = 11). FGF<145pg/mL was present in 19, and C4≥70nmol/L in 13 of the 25 patients with diarrhea. Three patients reported a number of weekly stools ≥21, all had FGF<145pg/ml, and two had C4≥70nmol/L. The subject who did not have a raised C4 had previously undergone cholecystectomy. Of the other two, one had a positive SeHCAT (12% 7-day retention), and the other had a Roux-en-Y gastric bypass, so SeHCAT testing was thought to be unnecessary for confirmation of the diagnosis of BAD. This patient had a good clinical response to cholestyramine. The number of stools per week did not correlate with NAFLD fibrosis score or Fibroscan stiffness (Table 3).

Median FGF19 in those with chronic diarrhea was less than in those without (73.0 vs 95.2 pg/mL), but this was not significant (p = 0.23)(Table 2). Median C4 was significantly raised for those with diarrhea (74.9 vs 35.4 nmol/mL, p = 0.0002). The odds ratio of a dysregulated FGF19 axis and chronic diarrhea occurring together was 6.2 (95%CI 2.1–18.2, p = 0.001).

### Effect of metformin and diabetes on chronic diarrhea and NAFLD severity

**Fig 2:**
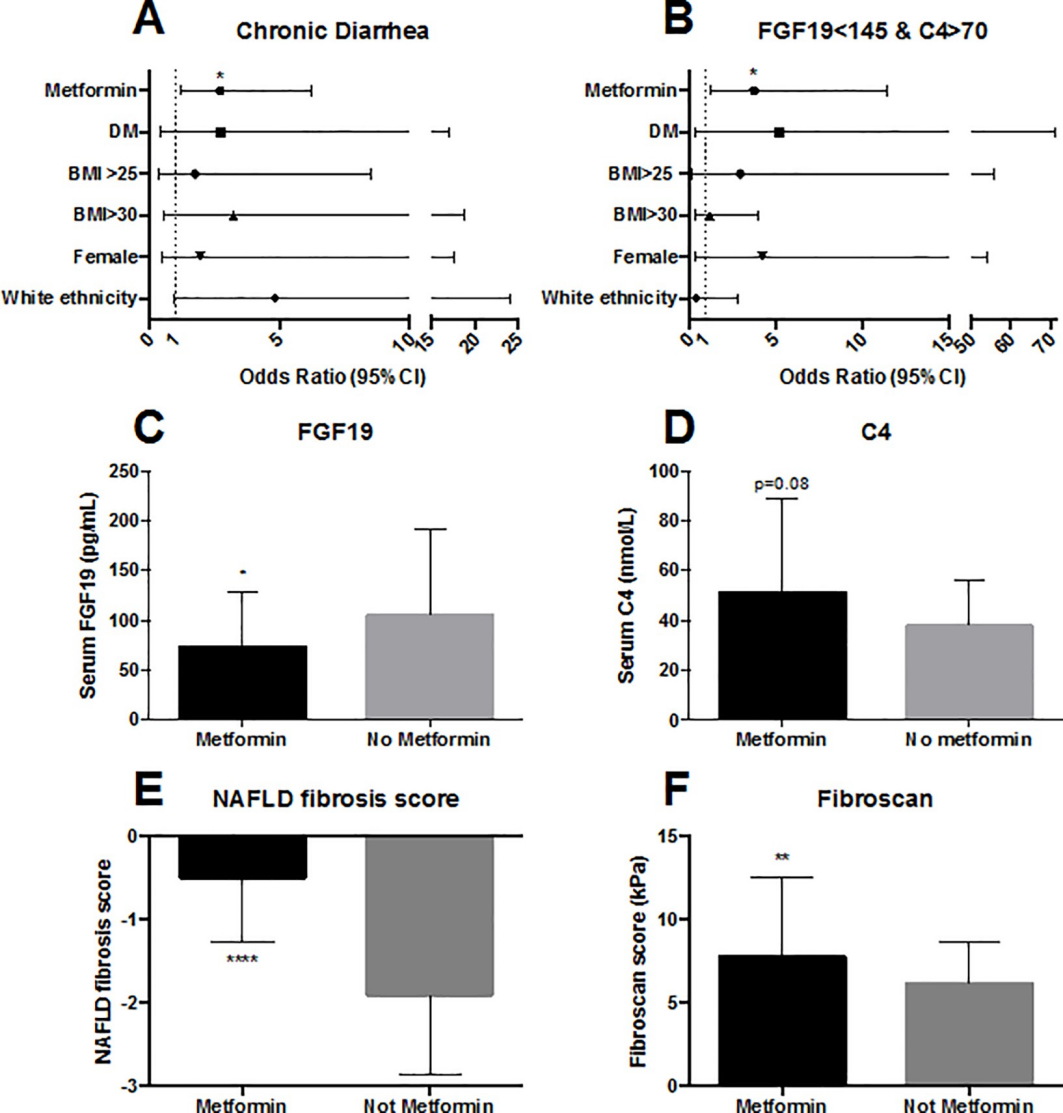
Effects of metformin use in NAFLD patients. Odds ratios (95%CI) of chronic diarrhea (A) and dysregulated FGF19 axis (B) by subgroup including metformin use, diabetes (DM), body mass index (BMI) >25 and >30, female gender and ethnicity. Median (IQR) FGF19 (C), C4 (D), NAFLD fibrosis score (E) and FibroScan score (F) by metformin use. *p<0.05, **p<0.01, ****p<0.00001.

The contribution of the effects of metformin to the findings are shown in Fig 2 which also shows the findings for the other subgroups (diabetes, raised BMI, gender and ethnicity). In the metformin group, 20/56 (36%) had diarrhea, compared to 12/71 (17%) in those not on metformin. When corrected for the other subgroup effects, the incidence of diarrhea was only significantly raised by the presence of metformin and/or diabetes; OR 2.7 (95%CI 1.19–6.25) and 2.6 (1.1–5.97), respectively, both p<0.05. As there were only 3 diabetic patients not taking metformin, and 3 non-diabetic patients taking metformin, these effects were indistinguishable from each other.

Median FGF19 was significantly lower on metformin 74.9 (IQR 56.7–128.2) vs 104.7pg/mL (IQR 68.4–183.7), p<0.05, (Fig 2C). Median C4 was higher on metformin, but not to a significant extent: 52.5 (IQR 28.1–87.2) vs 38.2 nmol/L (IQR 24.6–56.0), p = 0.08 (Fig 2D). Metformin was a significant risk factor for a dysregulated FGF19 axis, OR 3.7 (95%CI 1.2–11.4), p<0.05, but for bile acid diarrhea, as defined, the OR missed statistical significance, 3.2 (0.8–12.7), p = 0.10.

Metformin use was significantly associated with more severe NAFLD fibrosis score; median -0.50 (IQR -1.26 to 0.13), vs -1.90 (IQR -2.80 to -1.08), p<0.0001, (Fig 2E), and Fibroscan stiffness; median 7.8kPa (IQR 5.9–12.3) vs 6.2 (IQR 5.3–8.3), p<0.01, (Fig 2F). On biopsy staging, there were significantly more patients taking metformin who had cirrhosis (6 out of 7) than had steatohepatitis (1 out of 7), p<0.05.

### Effect of possible hepatic resistance

Hepatic resistance to FGF19 in obesity and insulin resistance has been described.[7, 25] To examine this effect in our cohort we looked at the C4/FGF19 ratio (high values reflecting loss of enterohepatic feedback) and the C4*FGF19 product (as a measure of hepatic resistance). There was a significant correlation of C4/FGF19 with fasting blood glucose (r_s_ = 0.21, p<0.05) but not with BMI, HDL cholesterol, triglycerides, NAFLD fibrosis score or Fibroscan. The C4*FGF19 product was significantly negatively correlated with ALT (r_s_ = -0.38, p<0.001) reflecting the low values of each described above. The product was also associated with triglycerides (r_s_ = 0.21, p<0.02) and NAFLD fibrosis score r_s_ = 0.18, p<0.04. Both ratios were associated with the presence of diarrhea (r_s_ = 0.3, p<0.002) showing the dominance of the C4 value in determining this.

## Discussion

In this prospective study of patients with NAFLD, we found a higher than expected incidence of chronic diarrhea, associated with increased bile acid production, as shown by raised C4 production. Lower FGF19 alone was not associated with NALFD severity in this cohort, but C4 was significantly associated with higher NAFLD fibrosis score, suggesting that dysregulation of the FGF19 axis is involved in NAFLD. We did not find that this effect was significantly reflected in more advanced disease, as shown by Fibroscan or liver biopsy results. However, metformin use in almost half of this cohort, and the presence of diabetes, were important contributing factors, and were associated with the presence of diarrhea and with more advanced disease.

The incidence of chronic diarrhea overall in this unselected, consecutive population of NAFLD was 25%. This appears to be a significantly increased rate but this remains uncertain without a control population. Diarrhoea was clearly more common (36%) in those on metformin and with diabetes, which comprise 44% of our cohort, but was present at 17% in those not on metformin and without diabetes. The figures we obtained are higher than expected and may reflect the composition of our specific cohort. The relationship with diabetes is well recognized however; for example, chronic diarrhea has been reported to be as prevalent as 15.6%, conferring an OR of 1.7 in a previous study.[26] Diarrhea is not usually recognized as a feature of NAFLD *per se*, although this was suggested from our previous retrospective study of chronic diarrhea patients, where, in the 52% who had BAD, an increased likelihood of steatosis was found.[20] In the general population, a prevalence of BAD of 1% has been estimated.[15]

The negative correlation of FGF19 with ALT has been described by others previously[11] and also lends validity to our previous finding, that SeHCAT retention percentage also has a negative correlation with ALT.[20] This finding does not extend to markers of fibrosis, and it is likely that multiple other steps will be involved. It is possibly a sample size error, since relatively few people with NAFLD progress to fibrosis.

The association of metformin with diarrhea is well described and is clearly of major relevance to our current findings. The possible mechanisms for this effect have been largely unknown until recently and bile acid diarrhea has never been considered. In our cohort, the effects of diabetes and metformin use on diarrhea were inseparable. The mechanisms underlying diarrhea in diabetes are multiple and include small bowel bacterial overgrowth, loss of α_2_ adrenergic stimulation and medications (usually metformin).[27] There is growing evidence that metformin interferes with bile acid metabolism. In a study where patients stopped metformin for around two weeks and then restarted, the total bile acid pool increased on metformin, and fecal bile acids decreased (although not significantly so) on metformin withdrawal.[28] Interestingly the authors conclude that part of metformin’s insulin sensitizing effect may be due to increased serum GLP-1, caused by increased stimulation by colonic bile acids of the G-protein coupled bile acid receptor TGR5 in enteroendocrine L-cells.

The principal mechanism of metformin action has been thought to be through activation of AMP-activated protein kinase (AMPK). AMPK is a regulator of FXR through phosphorylation and metformin reduced FGF15/19 in both human HepG2 cells and in mice.[29] In our cohort, median FGF19 was 30% lower on metformin and C4 was 37% higher. A recent paper looking at the effects of metformin found similar magnitude changes in FGF19 and C4 after only 3 days’ treatment.[30] Changes in the microbiome were described (specifically decreased *Bacteroides fragilis*), associated with increases in taurine and glycine conjugated ursodeoxycholic acids, which could be acting as FXR antagonists. The combination of those findings with ours suggests this as a potential mechanism for the bile acid diarrhea due to metformin found in our patients, and is an important new observation, meriting further study. However, we should stress that diarrhea was also commoner than expected in those of our patients who were not on metformin (incidence of 17%), and we do not know if there is a contribution of similar mechanisms in that group.

The relationship of symptoms of diarrhea to fecal bile acid loss is variable and there are many other pathophysiological causes and associated changes that may be implicated.[31, 32] If there was a shared major pathogenic mechanism behind NAFLD and BAD, we might expect the number of bowel movements to correlate with the severity of NAFLD. The presence of diarrhea was associated with a higher median NAFLD fibrosis score, but not higher Fibroscan scores, or fibrosis on biopsy. There were no significant correlations with these parameters and the daily number of stools.

There are many limitations to our study. These findings may only reflect the specific cohort of NAFLD patients that we identified in our clinic, and it is possible that many of the novel aspects are related to the diabetes and metformin treatment which was common in our cohort. More subjects would have provided clearer results and we were limited by the proportion of patients that returned stool diaries. Not all patients gave fasting blood samples. We could not be certain that all patients were avoiding recommended levels of alcohol consumption. Furthermore, it would have added interesting information if we had also collected stool and analyzed individual bile acids in this and in serum. Quantification of bile acids would have enhanced the study, and the interpretation of possible mechanisms, particularly in those patients who were on metformin, especially in view of the recent publication.[30] Another weakness of our study is the variability in histopathology reporting. Only 53% of our cohort had liver biopsies. We did not use a standardized reporting score, but we were limited to the reports written by different pathologists to assess the severity of the NAFLD.

A number of potential mechanisms linking NAFLD and BAD merit further consideration, in both groups of those patients taking, and not taking metformin, and will require further experimental study. We have not measured serum BA, but the increases shown in adolescent NASH patients by Jiao *et al*.[10] along with reduced FGF19 and increased C4, are in keeping with our initial hypothesis. An increase in the proportion of deoxycholic acid in blood was proposed as a possible mechanism, as this secondary BA is a weaker FXR agonist than chenodeoxycholic acid. This could also result in an antagonistic effect on the FXR signaling pathway. Changes in BA metabolism, producing more secondary BA, can be the result of dysbiosis in the colonic microbiome, but overall these results are inconsistent, with increased proportions of primary and not secondary BA in the feces having been described in NASH and in BAD.[9, 31] Also, obesity and insulin resistance have been shown to affect FGF19 sensing in the liver through increased miRNA-34a interference of the transcription of hepatic FGF19 co-receptor klotho-β.[25]Physiologically, this could produce hepatic FGF19 resistance, with the increased C4 levels reported here.

Further definitions of the BA/FXR/FGF19/FGFR4 axis phenotypes, and their relevance to disease, whether primary, caused by genetic polymorphisms, or secondary to acquired factors such as obesity or metformin, is an area of on-going research, both in BAD[32] and in NAFLD and NASH. These interactions, such as the associations we have described here, may help in the selection of phenotypic biomarkers, and in the selection of new therapeutic agents, such as FXR agonists, which could benefit a targeted group of patients with BAD and NAFLD or NASH.
